# A novel biocatalyst, *Enterobacter aerogenes* LU2, for efficient production of succinic acid using whey permeate as a cost-effective carbon source

**DOI:** 10.1186/s13068-020-01739-3

**Published:** 2020-05-29

**Authors:** Hubert Szczerba, Elwira Komoń-Janczara, Karolina Dudziak, Adam Waśko, Zdzisław Targoński

**Affiliations:** 1 Department of Biotechnology, Microbiology and Human Nutrition, University of Life Sciences in Lublin, 8 Skromna Street, 20-704 Lublin, Poland; 2 Chair and Department of Biochemistry and Molecular Biology, Medical University of Lublin, 1 Chodźki Street, 20-093 Lublin, Poland

**Keywords:** Succinic acid, Lactose, Whey permeate, *Enterobacter aerogenes*, Fermentation

## Abstract

**Background:**

Succinic acid (SA), a valuable chemical compound with a broad range of industrial uses, has become a subject of global interest in recent years. The bio-based production of SA by highly efficient microbial producers from renewable feedstock is significantly important, regarding the current trend of sustainable development.

**Results:**

In this study, a novel bacterial strain, LU2, was isolated from cow rumen and recognized as an efficient producer of SA from lactose. Proteomic and genetic identifications as well as phylogenetic analysis were performed, and strain LU2 was classified as an *Enterobacter aerogenes* species. The optimal conditions for SA production were 100 g/L lactose, 10 g/L yeast extract, and 20% inoculum at pH 7.0 and 34 °C. Under these conditions, approximately 51.35 g/L SA with a yield of 53% was produced when batch fermentation was conducted in a 3-L stirred bioreactor. When lactose was replaced with whey permeate, the highest SA concentration of 57.7 g/L was achieved with a yield and total productivity of 62% and 0.34 g/(L*h), respectively. The highest productivity of 0.67 g/(L*h) was observed from 48 to 72 h of batch fermentation, when *E*. *aerogenes* LU2 produced 16.23 g/L SA.

**Conclusions:**

This study shows that the newly isolated strain *E*. *aerogenes* LU2 has great potential as a new biocatalyst for producing SA from whey permeate.

## Background

Succinic acid (SA) is recognized as one of the top 10 most important C4-building blocks that can be produced from by-products and waste feedstock and converted into high-value commodities and specialty chemicals such as 1,4-butanediol (1,4-BDO), γ-butyrolactone (GBL) and tetrahydrofuran (THF) [1–3]. SA is also widely applied as an additive to food, pharmaceuticals, surfactants, solvents and detergents as well as during biodegradable polymer production [1, 4–6].

Until recently, succinate was commercially produced from *n*-butane through maleic anhydrate by a chemical process requiring the use of costly catalysts, and this process contributed to environmental problems [7, 8]. Therefore, because of pollution-reducing standards and global trends towards rational waste biomass management, the establishment of sustainable processes for the microbial production of SA from renewable feedstock has become a focal point of global interest [9–11].

At present, several biotech companies and joint ventures that have appeared over the past few years, including Bio-Amber/Mitsui, Myriant, Succinity (BASF/Corbion-Purac) and Reverdia (DSM/Roquette), have already initiated the microbial production of SA, which is considered one of the fastest-growing markets. The expected market size for bio-based succinate is estimated at 700,000 tons/year for 2020 [10–12].

Several renewable feedstocks are attractive for use as substrates in the microbial production of valuable bioproducts. Among them, cheese whey, a waste product of the dairy industry, is particularly interesting [13, 14]. Due to the high lactose content (> 80%) of whey permeate, which is recovered from cheese whey during the production of whey protein concentrate, this by-product can be an attractive, easy-to-use and low-cost substrate for succinate production [5, 15]. The price of whey permeate ranged from 0.4 to 0.62 euro/kg in 2019 (https://foodcom.pl/en/). Recently, a great deal of effort has been devoted to establishing biotechnological processes based on inexpensive, abundant and renewable raw substrates, including diverse lignocellulosic biomasses [10, 11, 16]. However, as reported by Cimini et al. [11], the biggest challenge is the extraction of fermentable sugars from complex cellulosic and hemicellulose matrices with high sugar yields. Szymanowska-Powałowska et al. [17] also claimed that the pretreatment of cellulose biomass is complex and costly. Importantly, the presence of toxic compounds contained in hydrolysate, including furfural and hydroxymethylfurfural (HMF), can affect the inhibition of cell growth and thus the production of succinate as well [10]. Meanwhile, whey permeate is an economically attractive feedstock, and it contains approximately 80% lactose as well as a high amount of micro- and macro-elements that lead to better cell growth [5, 15]. However, there is still little research on the effective use of this substrate as a sole carbon source for SA production.

Many bacterial strains have been screened and investigated for succinic acid production, including *Basfia succiniciproducens* [16], *Mannheimia succiniciproducens* [18], *Actinobacillus succinogenes* [7, 13, 19], *Anaerobiospirillum succiniciproducens* [20], *Corynebacterium glutamicum* [21] and recombinant *Escherichia coli* strains [22, 23]. These microorganisms are well studied and frequently used to produce succinate under anaerobic conditions [24]. Nevertheless, most of the identified strains have complex nutritional requirements and the ability to metabolize only simple carbon sources, primarily glucose. Meanwhile, there is still a need to screen strains that are able to use more complex carbon sources that are usually contained in by-products and waste feedstock. Thus, there are ongoing studies to find new succinate-producing strains with the abovementioned traits.

*Enterobacter aerogenes* has a fast growth rate and the ability to assimilate important carbon sources, including glucose, xylose, lactose, sucrose and glycerol. In addition to these attractive traits, this bacterium has low nutritional requirements and the ability to grow under both aerobic and anaerobic conditions, making it an attractive platform for producing bulk chemicals. Currently, *E. aerogenes* is being used to produce hydrogen, ethanol and 2,3-butanediol [25–27]. However, to our knowledge, studies on the use of this species for SA production from whey permeate have not yet been performed.

In this paper, a screen to identify succinic acid-producing bacteria from lactose was performed. Thorough MALDI-TOF/MS identification supported by 16S rDNA sequencing and phylogenetic analysis, the studied bacterium was classified as a strain of *Enterobacter aerogenes* named LU2. The effects of all the fermentation conditions, including the temperature, pH, yeast extract concentrations, inoculum size and initial substrate concentrations, were investigated by batch processing in 3-L fermenters. Finally, under optimal conditions, batch fermentations were performed with high concentrations of whey permeate. The results suggested that this new wild strain can be an efficient succinate producer.

## Results and discussion

### Identification of SA-producing microorganisms from lactose

To screen the succinate-producing microorganisms from lactose under anaerobic conditions, 50 bacterial isolates obtained from rumen samples were tested in bottle cultivation. Among these 50 isolates, 26 isolates were able to grow on lactose as the sole carbon source, while only one was able to secrete SA as the primary fermentation product. The SA spectrum in the fermentation broth completely overlapped with the standard substance. On the basis of the preliminary results, this strain was selected for further study for its capacity to produce SA by using whey permeate.

The morphology of the isolated strain was evaluated by electron microscope observation (Additional file 1: Fig. S1). Cells at the exponential growth phase were rod-shaped and 1.5–1.9 μm × 0.6–0.8 μm in size. The bacterial colonies on plates containing MHI medium were small, creamy, convex and shiny. The strain was Gram-negative and facultatively anaerobic. It had the ability to use various carbon sources, such as glucose, fructose, galactose, lactose, sucrose, maltose, xylose, cellobiose, sorbitol and glycerol, indicating its potential to produce other important bio-based products (Additional file 1: Table S1).

The identification of the selected strain based on MALDI-TOF/MS analysis showed that the strain was most similar to *Enterobacter aerogenes* LMG 2970 LMG, ATCC 13048T THL and 15282_1 CHB, with one score (2.233) pointing to probable species identification and three scores (2.369; 2.36; 2.349) pointing to highly probable species identification. To confirm the protein profiles obtained in MALDI-TOF/MS analysis and to make the identification more accurate, an additional approach was used.

Molecular identification based on sequencing the 16S rRNA-encoding gene and BLAST alignment of the sequences obtained against other DNA sequences deposited in the NCBI GenBank database indicated 99.9% shared similarity with *Klebsiella aerogenes* CAV1320 (NCBI accession number CP011574.1) and 99.86% shared similarity with both *Enterobacter aerogenes* EA1509E (NCBI accession number FO203355.1) and KCTC 2190 (NCBI accession number CP002824.1) [28, 29].

**Fig. 1:**
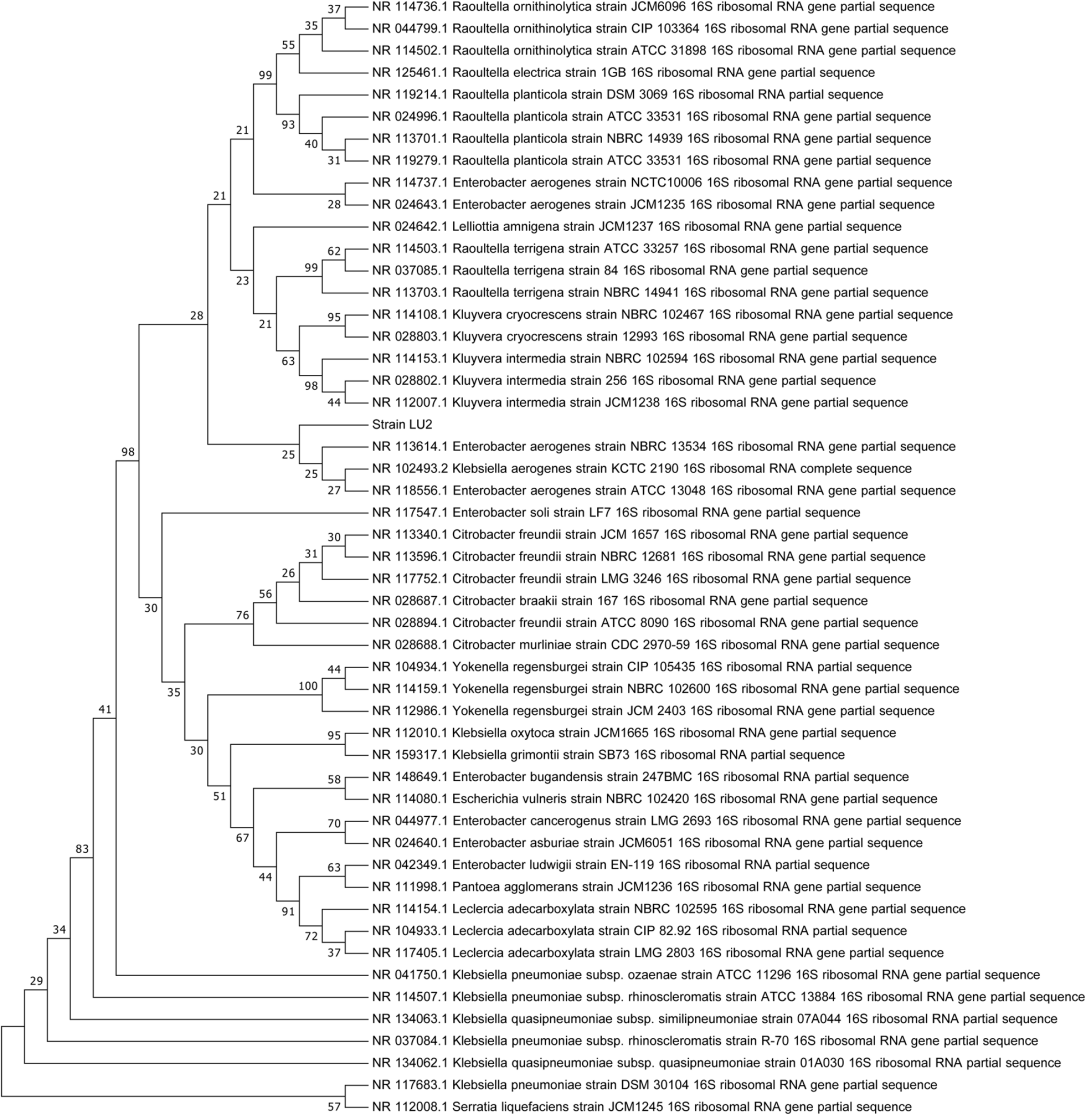
Phylogenetic tree constructed based on the comparison of 16S rDNA sequences showing the relationships among *Enterobacter aerogenes* LU2 and the other 50 strains belonging to the genera *Raoultella*, *Enterobacter*, *Lelliottia*, *Kluyvera*, *Citrobacter*, *Yokenella*, *Klebsiella*, *Escherichia*, *Pantoea*, *Leclercia* and *Serratia*. The tree was constructed using the maximum parsimony method. Bootstrap values (expressed as percentages of 1000 replications) are given at the nodes. The sequence accession numbers used for the phylogenetic analysis are given before the species name

Additionally, the phylogenetic tree constructed using the maximum parsimony method confirmed the proteomic and molecular identification, showing the strongest relationship of the isolated strain with *E*. *aerogenes* species, as depicted in the dendrogram in Fig. 1 [30, 31]. Considering the results, the newly isolated strain was classified as belonging to the *E*. *aerogenes* species and was named LU2.

Finally, strain *E*. *aerogenes* LU2 was deposited in the International Culture Collection of Industrial Microorganisms (CCIM) at the Institute of Agricultural and Food Biotechnology under the accession number KKP 2071p (Warsaw, Poland).

### Effects of temperature on cell growth and SA production

**Fig. 2:**
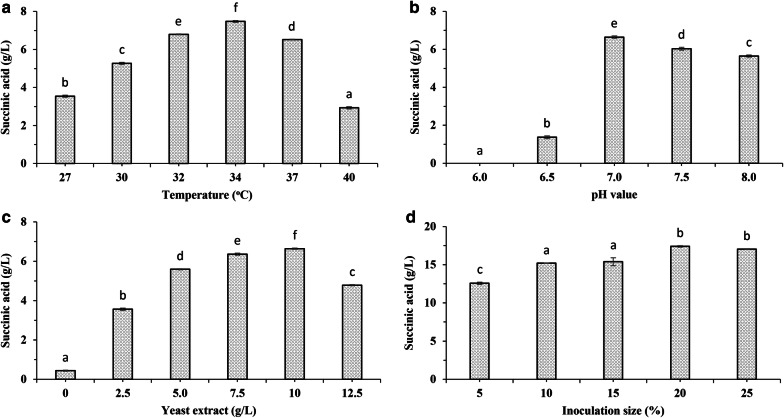
Effects of different fermentation conditions on succinic acid production by strain *Enterobacter aerogenes* LU2: **a** temperature; **b** pH value; **c** yeast extract; and **d** inoculum size. The values are the means of three independent samples. Bars represent the standard deviations. Different letters above the posts indicate significant differences (*p* ≤ 0.05) among the succinic acid concentration (g/L)

Temperature plays a crucial role in microbial growth and metabolism [32]. Podleśny et al. [33] indicated that the optimal temperature for cell growth by *Enterobacter* sp. LU1 was over the range of 27–34 °C, and the highest concentration of SA was achieved at 34 °C. Tajima et al. [34] also reported that 34 °C was the most suitable temperature for SA production by *E*. *aerogenes* AJ110637. Additionally, the temperature range of 30–33 °C was the most favourable for *Corynebacterium glutamicum* strains [21, 35]. Pinkian et al. [36] observed that 37 °C was the optimal temperature for SA production by two newly isolated strains, *Enterococcus durans* NS15-dA1 and *E*. *hirae* NS15-bA2. The same temperature was optimal for SA production by *Anaerobiospirillum succiniciproducens* [37], *Mannheimia succiniciproducens* [38] and *Basfia succiniciproducens* [16]. In general, most studies on the microbial production of SA have been conducted over a range of 30–37 °C. Therefore, the temperature optimization for strain *E*. *aerogenes* LU2 was performed under batch fermentation at 27 °C, 30 °C, 32 °C, 34 °C, 37 °C and 40 °C, with an initial lactose concentration of 20 g/L for 24 h in a 3-L bioreactor. The effects of different temperatures on the growth of strain *E*. *aerogenes* LU2 as well as SA production are shown in Fig. 2a and Additional file 1: Fig. S2. Strain *E*. *aerogenes* LU2 could grow normally from 27 to 40 °C. At 32 °C, 34 °C and 37 °C, the cell growth levels were largely comparable; however, the most favourable temperature was found to be 37 °C. At these temperatures, the strain reached the stationary growth phase after approximately 16 h. At 27 °C and 30 °C, the growth of strain *E*. *aerogenes* LU2 was slightly slower, while at 40 °C, the growth was significantly slower.

The relationship between the temperature within the range of 27–34 °C and the final SA concentration was clearly demonstrated. When the temperature was increased from 27 °C to 30 °C, 32 °C and 34 °C, the final titre of SA in the fermentation medium also increased, confirming the results obtained by Tajima et al. [34] and Podleśny et al. [33]. In addition, the lowest titre of SA was observed at 40 °C, probably due to the significantly lower activity of key enzymes at this temperature. According to statistical analysis, the mean final concentration of SA at 34 °C was significantly higher than the results obtained at other temperatures. Hence, the temperature of 34 °C was selected for further studies on strain *E*. *aerogenes* LU2.

### Effects of different pH values on cell growth and SA production

Environmental factors, particularly pH, can have a significant impact on the intracellular enzyme activity, substrate consumption rate and final titres of the target bioproducts [39]. This relevant factor affects both cell growth and the CO_2_/HCO_3_^−^ ratio during the fermentative production of SA [5]. Samuelov et al. [40] claimed that low pH values stimulate the production of succinate by increasing the activity of key enzymes engaged in PEP carboxykinase pathways. However, higher pH values result in better cell growth. Hence, identifying an optimal pH value is essential for further control of production as well as to ensure that the fermentation process is economically attractive. The optimal pH values for succinate production by anaerobic or facultative anaerobic bacterial strains were 6.7, 6.8, 6.5, 7.0, and 7.0 for *Actinobacillus succinogenes* ZT-130 ATCC 55617 [41], *Corynebacterium crenatum* J-2 [39], *Basfia succiniciproducens* BPP7 [16], *Enterococcus durans* NS15-dA1, and *E*. *hirae* NS15-bA2 [36], respectively.

To investigate the effect of pH on cell growth and SA production by strain *E*. *aerogenes* LU2, five pH values (6.0, 6.5, 7.0, 7.5, and 8.0) were maintained in the media during batch fermentation using 10% (v/v) NaOH and 20% (v/v) Na_2_CO_3_, with an initial lactose concentration of 20 g/L for 24 h at 34 °C in a 3-L bioreactor, as depicted in Fig. 2b and Additional file 1: Fig. S3. A slight difference in the final SA titre was observed at pH values of 7.0, 7.5 and 8.0; however, the highest concentration was noted at pH 7.0. Notably, at pH 6.5 or lower, the SA concentration was negligible, and a dramatic decrease in cell growth was observed, probably due to the innate inability of bacteria to assimilate carbon sources effectively in an acidic environment [42]. Based on statistical analysis, it has been shown that the mean final concentration of SA at pH 7.0 was statistically significantly higher than those obtained at other pH values. Considering the cell growth and the final SA concentration, the pH value of 7.0 was chosen as the most suitable and was used in further experiments.

### Effects of yeast extract concentration and inoculum size on SA production

The N source has a significant impact on both cell growth and succinate production [43]. Among the commonly used organic and inorganic N sources, yeast extract (YE) has been identified as one of the most favourable choices [7]. Apart from acting as an N source, it also contains trace metals and vitamins that have a positive effect on cell vitality and SA production [36]. YE concentrations (g/L) of 0, 2.5, 5.0, 7.5, 10.0, and 12.5 were investigated in batch fermentation with an initial lactose concentration of 20 g/L for 24 h at 34 °C and pH 7.0 in a 3-L bioreactor, as presented in Fig. 2c and Additional file 1: Fig. S4. When strain *E*. *aerogenes* LU2 was cultured in medium without YE, both cell growth and succinate production were negligible. When the YE concentration increased, the cell growth and succinate titre also increased. When the YE concentration reached 10 g/L, the highest cell growth as well as the highest succinate titre was obtained. An additional increase in the YE concentration led to slightly greater cell growth, but the succinate titre dropped by almost 30%. The statistical analysis indicated that the mean of the final concentration of SA obtained in the medium with 10 g/L YE was statistically significantly higher compared to the results obtained in the medium with any other studied YE concentration. Thus, a YE concentration of 10 g/L was chosen for further experiments.

The inoculation size plays an important role in SA production [32]. Hence, this factor was also investigated. Inoculum concentrations of 5%, 10%, 15%, 20% and 25% were tested in batch fermentation with an initial lactose concentration of 50 g/L for 48 h at 34 °C and pH of 7.0 in a 3-L bioreactor, as depicted in Fig. 2d. SA production was observed at all tested inoculum concentrations. When 5% of the inoculation size was used, the SA production was low. With an increased inoculum size, the SA concentration increased. The statistical analysis showed that the means of the final concentrations of SA were the highest in the media using 20% and 25% inoculum, and they were not significantly different. However, the optimal inoculum size was chosen as 20%, at which the highest titre of SA was obtained. Further increases in the inoculum size did not affect the higher SA titre in the fermentation medium.

### Effects of initial lactose concentration on cell growth and SA production

In many studies, the initial concentration of carbon source could affect both cell growth and metabolite production [5, 32, 43]. Thus, to increase the final concentration of SA, various initial lactose contents (60–140 g/L) were investigated for 96–144 h at 34 °C and pH 7.0 in a 3-L bioreactor.

**Fig. 3:**
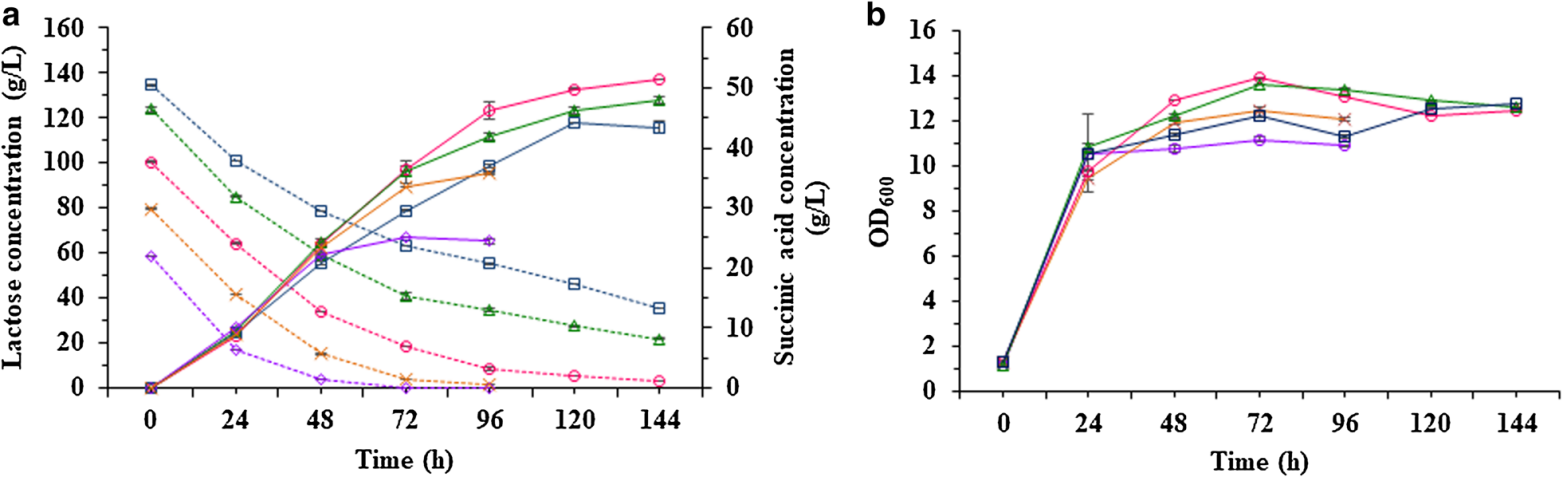
Effects of different lactose concentrations on the succinic acid production (**a**) and cell growth (**b**) of *Enterobacter aerogenes* LU2 under batch fermentation at 34 °C with stirring at 250 rpm in a 3-L bioreactor. Dashed lines and solid lines with the same colour and symbol represent the lactose, the corresponding succinic acid concentration and the cell growth as follows: 60 g/L lactose (purple), 80 g/L lactose (orange), 100 g/L lactose (pink), 120 g/L (green) lactose, and 140 g/L lactose (blue). The values are the means of three independent samples. Bars represent standard deviations

As shown in Fig. 3a, when the lactose concentration was below 100 g/L, SA production increased, along with an increase in the lactose content. The highest SA titre and yield of 51.35 g/L and 53%, respectively, were obtained at an initial lactose content of 100 g/L. In contrast, the final titre and yield of SA decreased when the lactose concentration was higher than 100 g/L. The lowest yield was obtained at a lactose content of 140 g/L.

The rapid consumption of lactose was observed within the first 24 h of batch fermentation at all the tested substrate contents. The high lactose utilization rate during this period was associated with an intensive increase in biomass formation (Fig. 3b). Importantly, even high concentrations of lactose (100–140 g/L) did not significantly affect the biomass within the first 24 h, indicating that *E*. *aerogenes* LU2 is resistant to high osmotic pressure and can tolerate high lactose contents during the fermentation process.

It is also worth noting that no glucose or galactose was detected in the fermentation medium during the batch process. This finding demonstrates that strain *E*. *aerogenes* LU2 has the ability to use lactose directly, and the lactose does not have to be broken down into simple sugars first.

### Batch fermentation with whey permeate

In the previous experiment, the highest concentration of SA was obtained when the lactose content in the fermentation medium was 100 g/L. To make the process more cost-effective, the possibility of using whey permeate derived from the local dairy plant instead of pure lactose was investigated.

**Fig. 4:**
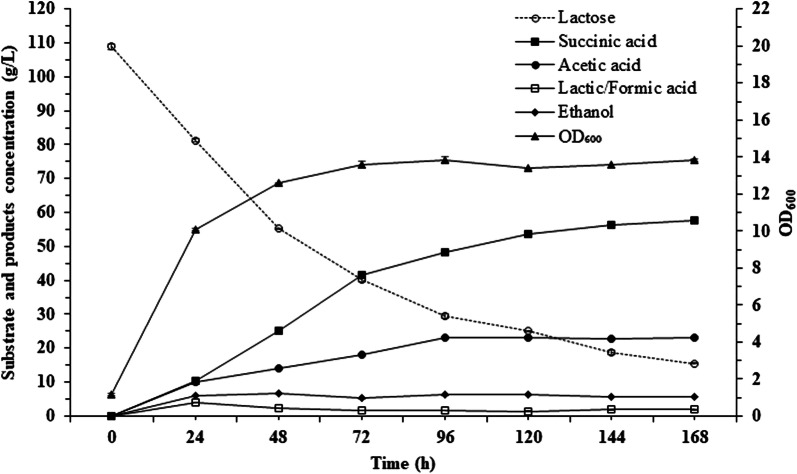
Production of succinic acid by *Enterobacter aerogenes* LU2 in batch fermentation. Lactose was replaced by whey permeate (which is approximately 83% lactose). The initial lactose concentration was approximately 100 g/L. The fermentation was performed at 34 °C with stirring at 250 rpm in a 3-L bioreactor. The pH was maintained at 7.0 using MgCO_3_. The values are the means of three independent samples. Bars represent standard deviations

As shown in Fig. 4, whey permeate had a positive effect on the fermentation parameters. The highest SA concentration and yield reached 57.7 g/L and 62%, respectively, with a total productivity of 0.34 g/(L*h). To our knowledge, this is the first report on such a high concentration of SA being produced by a wild-type strain of *Enterobacter* in batch fermentation. A rapid increase in the SA concentration was observed until 72 h when *E*. *aerogenes* LU2 produced 41.48 g/L SA, with a total productivity of 0.58 g/(L*h). However, the highest productivity of 0.67 g/(L*h) was noted between 48 and 72 h of batch fermentation, when 16.23 g/L SA was produced. After 72 h of fermentation, the productivity of the process had dropped, probably due to the accumulation of other by-products (primarily acetic acid), which can inhibit succinate production. However, it is worth noting that the final concentration of by-products decreased slightly compared to their concentration when pure lactose was the primary carbon source. Despite that concern, attempted genetic modification of the strain should be performed to reduce the concentration of by-products in the future. Guo et al. [32] also indicated that a shortage of nutrients may result in decreased production, although, as shown in Fig. 4, *E*. *aerogenes* LU2 reached the logarithmic growth phase in 72 h, and the OD600 value was almost constant until the end of the fermentation process. Notably, when pure lactose was applied, *E*. *aerogenes* LU2 reached the logarithmic growth phase at the same time, but during the later period of fermentation, a decreased OD600 was observed. Better growth of *E*. *aerogenes* LU2 on whey permeate is probably caused by the additional source of trace metals as well as mineral salts contained in this feedstock, which can provide cells with vitality.

Compared to other well-known and high-level SA bacterial producers, the highest titre of SA produced from whey was obtained for *E*. *aerogenes* LU2 (Table 1). In turn, the fermentation time for *E. aerogenes* LU2 was longer compared to that for other strains shown in Table 1. However, it is worth noting that this is the first report presenting the initial optimization of the process, which was to determine the predisposition of the strain in terms of the highest daily SA production, the highest SA titre, and the possibility of adapting the strain for continuous fermentation. The results obtained provide hope for future optimization of this bioprocess, including shortening the fermentation time while maintaining a high SA titre and obtaining a much higher productivity.

**Table 1:** Production of succinic acid from whey in batch, fed-batch and continuous fermentations by different wild-type bacterial strains

Strain	Substrate	Fermentation conditions	Fermentation type	Fermentation time (h)	Final titre (g/L)	Yield (g/g)	Productivity (g/L*h)	References
*Actinobacillus succinogenes* 130Z	Whey	Anaerobic	Batch	48 (± 1)	27.9	0.43	0.58	[5]
*Mannheimia succiniciproducens* MBEL55E	Whey	Anaerobic	Batch	11 (± 1)	13.4	0.71	1.18	[47]
	Whey	Anaerobic	Continuous	–	6.4	0.69	3.9	
*Anaerobiospirillum succiniciproducens* ATCC 29305	Whey	Anaerobic	Batch	65 (± 1)	15.5	0.93	0.24	[20]
	Whey	Anaerobic	Fed-batch	34 (± 3)	34.7	0.91	1.02	[40]
	Whey	Anaerobic	Continuous	–	19.8	0.64	3	
	Whey	Anaerobic	Continuous	–	14.3	0.71	3.3	[48]
*Enterobacter aerogenes* LU2	Whey permeate	Anaerobic	Batch	168 (± 1)	57.7	0.62	0.34	This study

## Conclusions

In this study, a newly isolated SA-producing strain named *E*. *aerogenes* LU2 was reported. *E*. *aerogenes* LU2 can effectively convert lactose into SA under anaerobic conditions. The optimal temperature for succinate production was found to be 34 °C, and the optimal pH was 7.0. The feasibility of using whey permeate as an economically attractive substitute for pure lactose has been demonstrated. Through batch fermentation in a 3-L stirred bioreactor, 57.7 g/L SA was produced, with a yield of 62%. The results obtained here suggest that *E*. *aerogenes* LU2 has the potential to be a new and efficient platform for producing SA.

## Materials and methods

### Chemicals

Lactose (≥ 99.0% purity) and succinic acid (≥ 99.0% purity) were purchased from Sigma-Aldrich (Saint Louis, Missouri, USA). The whey permeate used in this study was kindly provided by Bempresa (Ostrów Lubelski, Poland) and had the following composition: lactose ≥ 83%, proteins 3–5%, fats ≤ 1%, water ≤ 4%, ashes ≤ 8.5%. The other chemicals were of reagent grade and were from Oxoid (Basingstoke, Hampshire, England), BTL (Warszawa, Poland) or POCH (Gliwice, Poland) unless otherwise specified.

### Screening to identify bacteria capable of producing SA from lactose

In our previous study, samples from the rumen of ruminants were collected, and bacteria were isolated by bacterial enrichment and selective culture [33]. All the resulting bacterial isolates were deposited in our laboratory’s collection.

In the present study, a screening procedure aimed at selecting bacterial isolates that have the capacity to produce SA from lactose under anaerobic conditions was performed as follows. The bacterial isolates were maintained frozen at − 80 °C with 20% (w/w) added glycerol. The cells were grown under anaerobic conditions in 100-mL bottles with gas-tight butyl rubber stoppers filled halfway with brain heart infusion (BHI) medium (Oxoid, Basingstoke, Hampshire, England) containing the following components (g/L): brain infusion solids (12.5), beef heart infusion solids (5.0), protease peptone (10.0), glucose (2.0), sodium chloride (5.0), and disodium phosphate (2.5), pH 7.4, and cultured for 22 h at 37 °C. The bacterial cultures were then used to inoculate the fermentation medium (5% (v/v)), which contained the following (g/L): lactose (100), yeast extract (10), K_2_HPO_4_ (1), MgSO_4_ × 7H_2_O (0.2), and CaCl_2_ (0.5). Solid MgCO_3_ (60 g/L) was added to the media to provide indirect CO_2_ and serve as a pH buffer of the fermentation broth [7]. The carbon (C) and nitrogen (N) sources were sterilized separately for 20 min at 121 °C before use, and then they were mixed together aseptically [44].

All screening cultivations were conducted in 100-mL bottles with gas-tight butyl rubber stoppers (each containing 50 mL of fermentation medium) in a rotary shaker (150 rpm) (Minitron Incubator Shaker, Infors AG, Switzerland) for 144 h at 34 °C. The experiments were performed with at least three full biological repeats.

### Analytical methods

After anaerobic bottle cultivation, the removal of MgCO_3_ from the fermentation broth was performed by diluting the sample 1:1 with 7% HCl (v/v) [15]. Cell growth was monitored by measuring the absorbance at 600 nm (OD600_nm_) using a SmartSpec Plus Spectrophotometer (Bio-Rad, Hercules, USA). Broth samples were prepared by centrifugation at 10,000 × g for 10 min. After that, the resulting supernatants were filtered with a 0.22 μm membrane syringe filter (Millipore, Burlington, USA) and diluted with deionized water (1:1). The samples were analysed using a high-performance liquid chromatography (HPLC) system (Gilson, Middleton, USA) equipped with a UV–VIS DAD detector (Gilson, Middleton, USA), refractive index detector (RI) (Knauer, Berlin, Germany) and ion exchange column (Aminex HPX-87H) (Bio-Rad, Hercules, USA) at 42 °C with 0.03 M sulfuric acid as the mobile phase at a flow rate of 0.5 mL/min. The injection volume was 10 μL, and the total runtime was 30 min. An analysis of the chromatographic data was performed using Gilson Unipoint 2.0 (Gilson, Middleton, USA) and Chromax software (POL-LAB, Warszawa, Poland).

### Proteomic identification of the isolated strain based on MALDI-TOF/MS analysis

The screened bacterial isolate was grown anaerobically at 37 °C for 24 h on BHI medium (Oxoid, Basingstoke, Hampshire, England) with 2% agar (BTL, Łódź, Poland). After that, four single colonies of the screened isolate were harvested and prepared separately for analysis according to a standard procedure recommended by the manufacturer (Bruker Daltonics, Bremen, Germany) as described previously by Paściak et al. [45]. A sample of the extracted proteins was transferred onto an MTP 384 ground steel target plate and overlaid with α-cyano-4-hydroxycinnamic acid (HCCA) matrix solution suspended in acetonitrile (ACN) and trifluoroacetic acid (TFA), as recommended by the manufacturer. All measurements were performed with an UltrafleXtreme MALDI-TOF mass spectrometer (Bruker Daltonics, Bremen, Germany). The mass spectra were collected in positive linear mode over a mass range of 2.000–20.000 Da using FlexControl 3.1 (Bruker Daltonics, Bremen, Germany) software and were then compared with the reference library by MALDI Biotyper 3.1 (Bruker Daltonics, Bremen, Germany) software. Prior to the measurements, the mass spectrometer was calibrating using a Bruker Bacterial Test Standard (BTS) (Bruker Daltonics, Bremen, Germany) containing an extract of *E*. *coli* DH5-alpha (RNase A, myoglobin proteins). According to the manufacturer’s instructions, the identification criteria were dependent on a confidence interval of the obtained score value and were as follows: 0–1.699 (not reliable identification), 1.7–1.999 (probable genus identification), 2–2.299 (secure genus identification, probable species identification), and 2.3–3 (highly probable species identification).

### Genetic identification of the isolated strain based on 16S rDNA sequencing

To confirm the MALDI-TOF/MS-based identification results, 16S rDNA nucleotide sequencing of the screened isolate was performed. For quality assurance, genomic DNA was extracted from a pure culture of a single bacterial isolate that was previously passaged twice. Total genomic DNA isolation was performed using a Genomic Micro AX Bacteria + Gravity kit (A&A Biotechnology, Gdynia, Poland) according to the manufacturer’s instructions (2017). Genomic DNA was used to amplify the 16S rRNA-encoding gene enzymatically using the primers 27F (5′-AGA GTT TGA TCC TGG CTC AG-3′) and 1492R (5′-TAC GGY TAC CTT GTT ACG ACT T-3′), and an amplicon measuring approximately 1390 bp was produced [28]. The reaction was performed in a total volume of 25 μL using 2x PCR Master Mix (Thermo Fisher Scientific, Waltham, USA) with 20 pmol of each primer (Genomed, Warszawa, Poland) and 60 ng of DNA template using a LabCycler (SensoQuest GmbH, Göttingen, Germany). The PCR thermal conditions were as follows: denaturation at 95 °C for 3 min, 30 cycles of 15 s at 95 °C, 15 s at 58 °C, 2 min at 72 °C, and a final extension at 72 °C for 8 min. The amplification products were separated by electrophoresis on 2% (w/v) agarose gels (EURx, Gdańsk, Poland) stained with SimplySafe (EURx, Gdańsk, Poland) in 1× TBE for 1.5 h at 120 V. The DNA bands were visualized and archived using a GelDoc XR + Gel Documentation System (Bio-Rad, Hercules, USA) and Image Lab software (Bio-Rad, Hercules, USA). The sizes of the amplicons were determined with a GeneRuler 100 bp + DNA ladder (Thermo Fisher Scientific, Waltham, USA). The amplicon was purified using a Clean-Up kit (A&A Biotechnology, Gdynia, Poland). DNA sequencing was performed using a BigDye Terminator v. 3.1 Cycle Sequencing Kit (Thermo Fisher Scientific, Waltham, Poland) and capillary sequencer system, namely, Applied Biosystems 3130XL (Applied Biosystems, Foster City, USA). The 16S rDNA sequence obtained was subjected to visual inspection and editing after sequencing. Finally, the resulting sequence was compared against homologous sequences deposited in the NCBI GenBank database using the NCBI BLAST algorithm [29].

### Phylogenetic analysis

The 16S rDNA sequences of strain LU2 and other strains belonging to different genera within the *Enterobacteriaceae* family were used to perform multiple nucleotide alignments with the ClustalW algorithm [46] implemented in MEGA X [31]. The nucleotide sequences were retrieved from the NCBI GenBank 16S ribosomal RNA sequences database. The phylogenetic tree was inferred with the maximum parsimony method using a max–mini branch-and-bound algorithm in MEGA X software, with bootstrap values based on 1000 replicates [30, 31].

### Optimization of SA production by batch fermentation in a stirred bioreactor

The SA production by *E*. *aerogenes* LU2 was optimized as follows. The *E*. *aerogenes* LU2 strain was maintained frozen at − 80 °C with 20% (w/w) added glycerol. The inoculated culture was grown semi-anaerobically for 19–22 h in BHI (Oxoid, Basingstoke, Hampshire, England) medium in a rotary shaker (Minitron Incubator Shaker, Infors AG, Switzerland) at 37 °C and 150 rpm. The screened strain was then cultured in fermentation medium whose composition was different, depending on the fermentation conditions that have been studied.

The optimization of the temperature (27–40 °C) was conducted in batch fermentation for 24 h with medium containing (g/L) lactose (20), yeast extract (10), K_2_HPO_4_ (1), MgSO_4_ × 7H_2_O (0.2), CaCl_2_ (0.5), and MgCO_3_ (20). Subsequently, the optimal pH value (6.0–8.0) was investigated in batch fermentation for 24 h at 34 °C with the medium of the same composition. The medium used to optimize the YE concentration contained (g/L) lactose (20), yeast extract (0–12.5), K_2_HPO_4_ (1), MgSO_4_ × 7H_2_O (0.2), CaCl_2_ (0.5), and MgCO_3_ (20), and batch fermentation was conducted for 24 h at 34 °C and pH 7.0. The medium used for testing optimal inoculum concentrations (5–25%) contained (g/L) lactose (50), yeast extract (10), K_2_HPO_4_ (1), MgSO_4_ × 7H_2_O (0.2), CaCl_2_ (0.5), and MgCO_3_ (20), and batch fermentation was conducted for 48 h at 34 °C and pH 7.0. Finally, for the optimization of the optimal initial lactose concentration (60–140 g/L), the medium contained (g/L) lactose (60–140), yeast extract (10), K_2_HPO_4_ (1), MgSO_4_ × 7H_2_O (0.2), and CaCl_2_ (0.5), and batch fermentation was performed for 96–144 h at 34 °C and pH 7.0.

All batch fermentations were conducted in a 3-L Sartorius Biostat A plus bioreactor with a working volume of 2 L (Sartorius Stedim, Melsungen, Germany). To investigate the optimal temperature, pH value and YE concentration, the inoculum size was 5%, while for testing the optimal lactose concentration, 20% inoculum was used. For optimization of the pH value, YE concentration and inoculum size, the pH was maintained using 10% (v/v) NaOH and 20% (v/v) Na_2_CO_3_, while to investigate the optimal lactose concentration, the pH was maintained using MgCO_3_ [7, 33]. When whey permeate was used, the added amount was based on the corresponding lactose content. The C and N sources were separately sterilized for 15 min at 121 °C. The stirrer rate was 250 rpm in all cultures. During cultivation, 10–40 mL samples were collected from the bioreactor at regular time intervals to determine cell growth, lactose consumption and metabolite production.

### Fermentation parameters

The SA yield (*Y*_SA_) was expressed as grammes of SA produced per gramme of lactose consumed, and the volumetric SA productivity (*Q*_SA_) was expressed as grammes of SA produced per litre per hour. The *Y*_SA_ and *Q*_SA_ were calculated using Eqs. (1) and (2), respectively, as follows:

1 $$Y_{\text{SA}} \left( \% \right) = \frac{{\left[ {\text{SA}} \right]}}{\left[ L \right]} \times 100\%,$$

2 $$Q_{\text{SA}} = \frac{{\left[ {\text{SA}} \right]}}{\left[ t \right]},$$

where “SA” is the succinate concentration in the fermentation medium (g/L); “*L*” is the amount of lactose that was consumed during fermentation (g/L); and “*t*” is fermentation duration (h).

### Statistical analyses

One-way ANOVA followed by Tukey’s post hoc test for pairwise comparison of means (at *p* ≤ 0.05) was used to assess the difference in SA production under different fermentation conditions and on various fermentation media. Statistical analysis was performed using the Statistica 13.1 statistical software package (StatSoft, Kraków, Polska).

## Supplementary information

**Additional file 1: Fig. S1.** Scanning electron microscope micrograph of *Enterobacter aerogenes* LU2. **Fig. S2.** Cell growth of *Enterobacter aerogenes* LU2 at different temperatures. **Fig. S3.** Cell growth of *Enterobacter aerogenes* LU2 at different pH of fermentation medium. **Fig. S4.** Cell growth of *Enterobacter aerogenes* LU2 at different yeast extract concentrations. **Table S1.** Cell growth of *Enterobacter aerogenes* LU2 on different carbon sources.

## Data Availability

All data generated or analysed during this study are included in this published article [and its Additional files].

## Abbreviations

SA: Succinic acid
1,4-BDO: 1,4-Butanediol
GBL: γ-Butyrolactone
THF: Tetrahydrofuran
HMF: Hydroxymethylfurfural
C: Carbon
N: Nitrogen
YE: Yeast extract
BHI medium: Brain heart infusion medium
HPLC: High-performance liquid chromatography
