# Supplementary material for: A novel biocatalyst, Enterobacter aerogenes LU2, for efficient production of succinic acid using whey permeate as a cost-effective carbon source
Source: Biotechnol Biofuels. 2020 May 29;13:96. doi: 10.1186/s13068-020-01739-3 (PMC7257193; doi:10.1186/s13068-020-01739-3)
Supplement: Supplementary file 1 — Additional file 1: Fig. S1. Scanning electron microscope micrograph of Enterobacter aerogenes LU2. Fig. S2. Cell growth of Enterobacter aerogenes LU2 at different temperatures. Fig. S3. Cell growth of Enterobacter aerogenes LU2 at different pH of fermentation medium. Fig. S4. Cell growth of Enterobacter aerogenes LU2 at different yeast extract concentrations. Table S1. Cell growth of Enterobacter aerogenes LU2 on different carbon sources. [file 13068_2020_1739_MOESM1_ESM.docx]

**Additional materials for**

**A novel biocatalyst, *Enterobacter aerogenes* LU2, for efficient production of succinic acid using whey permeate as a cost-effective carbon source**

Hubert Szczerba^1^*, Elwira Komoń-Janczara^1^, Karolina Dudziak^2^, Adam Waśko^1^, Zdzisław Targoński^1^

^1^Department of Biotechnology, Microbiology and Human Nutrition, University of Life Sciences in Lublin, 8 Skromna Street, 20-704 Lublin, Poland

^2^Chair and Department of Biochemistry and Molecular Biology, Medical University of Lublin, 1 Chodźki Street, 20-093 Lublin, Poland

***Corresponding author:**

E-mail address: [hubert.szczerba@up.lublin.pl](about:blank) (Hubert Szczerba)

Tel: (0048) 81 462 34 02; Fax: (0048) 81 462 34 00

Department of Biotechnology, Microbiology and Human Nutrition,

University of Life Sciences in Lublin, 8 Skromna Street, 20-704 Lublin, Poland

**
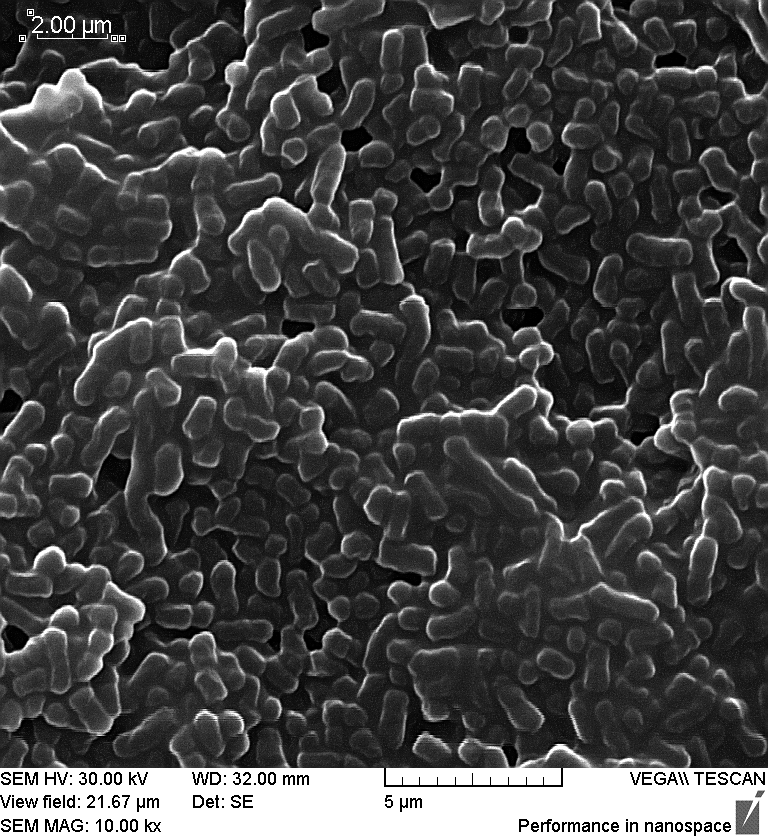
**

**Fig. S1.** Scanning electron microscope micrograph of *Enterobacter aerogenes* LU2.

**
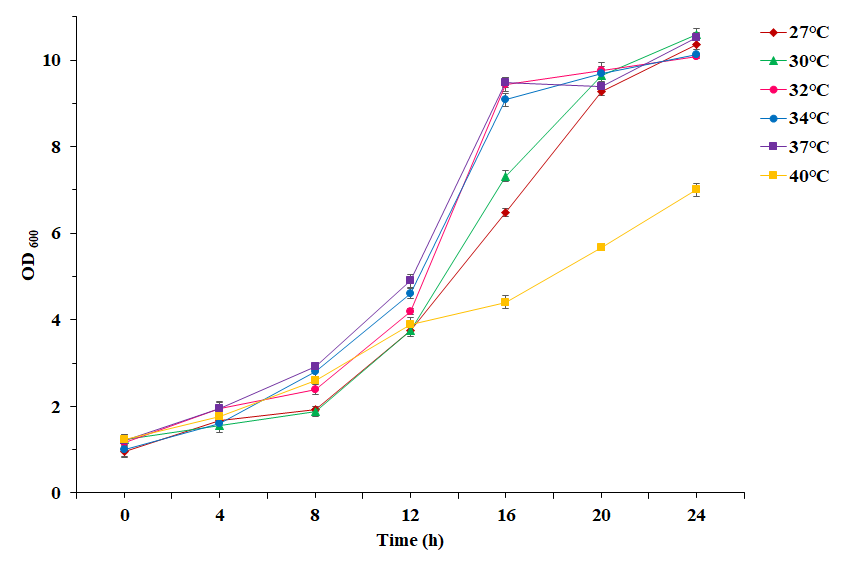
Fig. S2.** Cell growth of *Enterobacter aerogenes* LU2 at different temperatures.

**
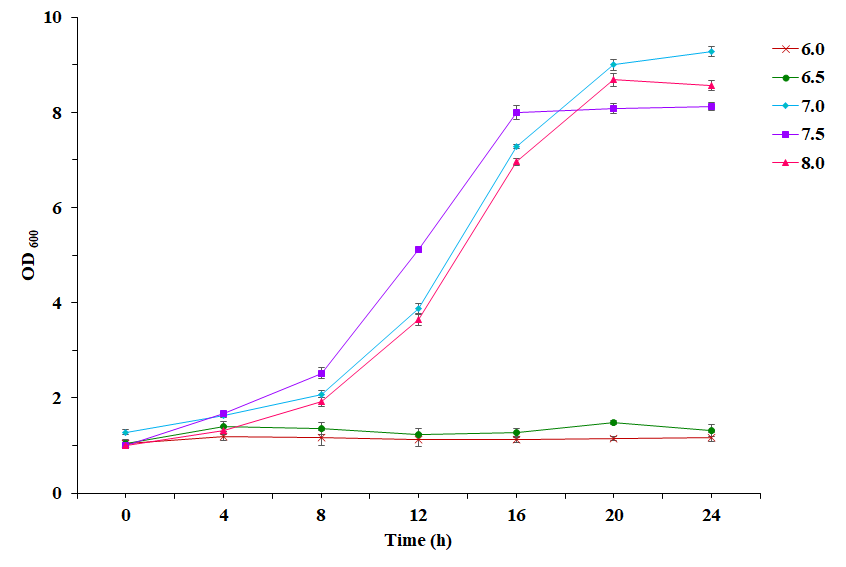
Fig. S3.** Cell growth of *Enterobacter aerogenes* LU2 at different pH of fermentation medium.

**
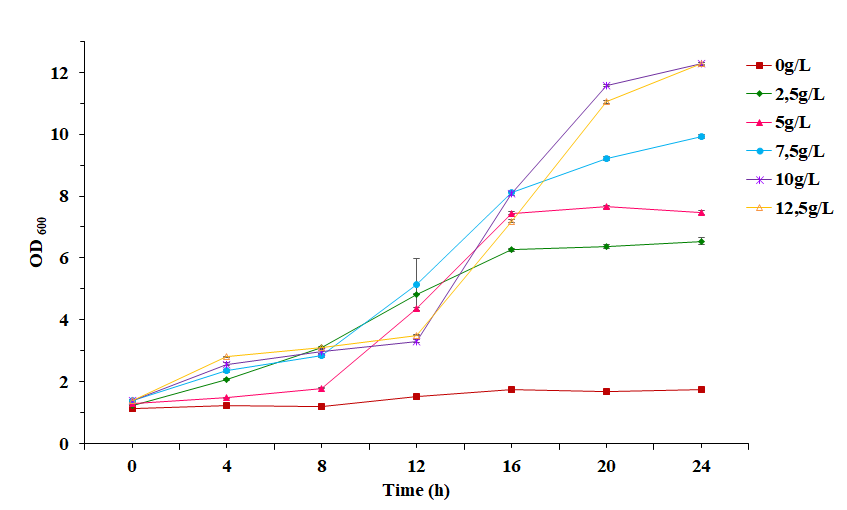
**

**Fig. S4.** Cell growth of *Enterobacter aerogenes* LU2 at different yeast extract concentrations.

**Tab. S1.** Cell growth of *Enterobacter aerogenes* LU2 on different carbon sources.

| Carbon source | OD_600_ |
| --- | --- |
| Glucose | 4.24 ± 1.61 |
| Fructose | 7.35 ± 0.21 |
| Galactose | 7.6 ± 0.85 |
| Xylose | 8.47 ± 0.24 |
| Lactose | 15.06 ± 0.08 |
| Maltose | 9.2 ± 0.28 |
| Sucrose | 8.62 ± 0.31 |
| Cellobiose | 11.45 ± 0.35 |
| Glycerol | 4.55 ± 0.66 |
| Sorbitol | 8.35 ± 0.21 |

**Description of experiment**

The ability to metabolize various carbon sources has been verified in batch fermentation. The strain was maintained frozen at -80°C with 20% (w/w) glycerol added. The inoculum was cultured under anaerobic conditions in 100 mL bottles with gas-tight butyl rubber stoppers filled halfway by BHI (Oxoid, UK) medium for 22 h at 37°C. Bacterial culture was then used to inoculate fermentation medium (5% (v/v)) with the following composition (g/L): carbon source (100); yeast extract (10); K_2_HPO_4_ (1), MgSO_4_ x 7H_2_O (0,2); CaCl_2_ (0,5); MgCO_3_ (60). MgCO_3_ was added as a pH buffer of the fermentation broth. Carbon (C) and nitrogen (N) sources were sterilized separately for 20 min at 121°C before use and then were mixed together aseptically. The experiment was carried out in 100 mL bottles (each containing 50 mL of fermentation medium) with gas-tight butyl rubber stoppers in a rotary shaker (150 rpm) (Minitron Incubator Shaker, Infors AG, Switzerland) for 144 h at 34°C. Removal of MgCO_3_ was performed by diluting the sample 1:1 with 7% HCl (v/v), and cell growth was verified by measuring the absorbance at 600 nm (OD600_nm_) using a SmartSpec Plus Spectrophotometer (Bio-Rad, Hercules, USA).
